# A Rare Case of Creutzfeldt–Jakob Disease With Alcohol Use Disorder and Review of Literature

**DOI:** 10.7759/cureus.24812

**Published:** 2022-05-07

**Authors:** Abraham Joseph, Hisham Mushtaq, George Zakhia, Jonathan Rohde, Adria Whiting, Abbas B Jama, Anwar Khedr, Nitesh K Jain, Syed Anjum Khan

**Affiliations:** 1 Hospital Medicine, Mayo Clinic Health System, Fairmont, USA; 2 Critical Care Medicine, Mayo Clinic Health System, Mankato, USA; 3 Neurology, Mayo Clinic Health System, Mankato, USA; 4 Family Medicine, Mayo Clinic Health System, Fairmont, USA

**Keywords:** scjd, cjd, alcohol disorder, acute dementia, prion disease, sporadic creutzfeldt–jakob disease, creutzfeldt–jakob disease

## Abstract

Sporadic Creutzfeldt-Jakob disease (CJD) is a rare neurodegenerative disorder, accounting for a majority of the sporadic prion disease burden. This disorder rapidly progresses and is often fatal with no known cure. Initial diagnosis may be delayed due to its varied presentations, which can include psychiatric changes (behavioural and mood variances), visual and auditory hallucinations, cerebellar dysfunction, and pain, occurring in isolation in many cases. Due to the nonspecific complaints, accurate diagnosis can be challenging. CJD exhibits symptoms similar to other neuropsychiatric illnesses; however, only a few reports have been published concerning the association between CJD and alcohol-related illnesses. This case report demonstrates the challenge of diagnosing this disorder early in the clinical course given the variable presentation, especially in a patient with a history of an alcohol use disorder, falls, and cognitive decline.

## Introduction

Sporadic Creutzfeldt-Jakob disease (CJD) is a prion disease that is a group of heterogenous and phenotypically diverse disorders occurring in 1-1.5 cases per 1,000,000 per year worldwide [1]. It is caused by an abnormal accumulation and misfolded prion proteins that lead to neuronal death [2-4]. These neuronal changes lead to a spongiform appearance of the brain, which can be seen in magnetic-resonance imaging (MRI). The misfolded proteins termed PrPsc are resistant to proteases and can facilitate rapid conversion of normal PrP to PrPsc, which leads to active disease progression [2]. CJD is untreatable, as it always results in death. It is generally fatal within a year of symptom onset, with a median duration of five to six months [5,6]. It is possible for any patient suffering from a neuropsychiatric disorder to develop CJD. Therefore, CJD complicated by a neuropsychiatric disorder may be difficult to identify from the disorder itself. We present a rare case of CJD with alcohol use disorder.

## Case presentation

A 74-year-old man with a significant history of alcohol use disorder and diabetes mellitus type 2 presented for evaluation of progressive cognitive decline, frequent falls, and tremors, which had been ongoing for about six months. The patient’s family noted progressive cognitive decline starting approximately six months prior to presentation. The family noted he was forgetting and misplacing items, as well as missing important appointments. At the initial presentation, he was fully functional with regards to his instrumental activities of daily living (IADLs) and activities of daily living (ADLs). The patient was noted to have a slow gait, tremors in the upper extremities, and frequent falls, which were attributed to a history of alcohol use, but on further evaluation, the patient was noted to have cut down his drinking to about 4-6 units of alcohol per week. The patient reported that he no longer drove automobiles after he was involved in a motor vehicle accident. His family described the incident as "his leg locked on the gas pedal, causing him to drive on to his front yard, then when backing out, his leg locked again, causing him to crash into a tree." His exam was concerning for cognitive decline, scoring 23/28 on his Kokmen Short Test of Mental Status, with major deficiencies in his short-term memory, calculation, and construction. Eye movements showed a full range of movements with slowed saccades. He had both resting and intentional tremors, mainly affecting the upper extremity more on the left, with rigidity in his upper extremity and bradyphrenia. His gait was unstable, wide-based with decreased arm swing mainly over the left side.

**Figure 1:**
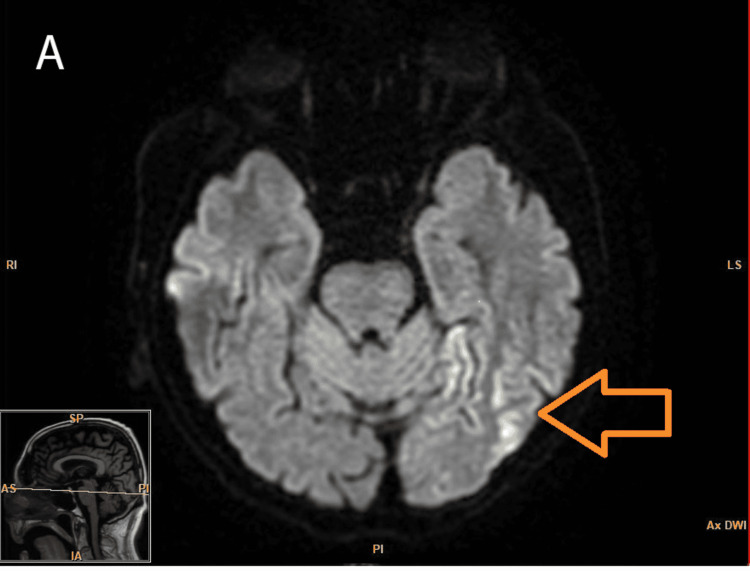
Axial diffusion-weighted MRI showing hyperintensities Bilateral cortical high signal intensities, more prominent on the left side (arrow)

**Figure 2:**
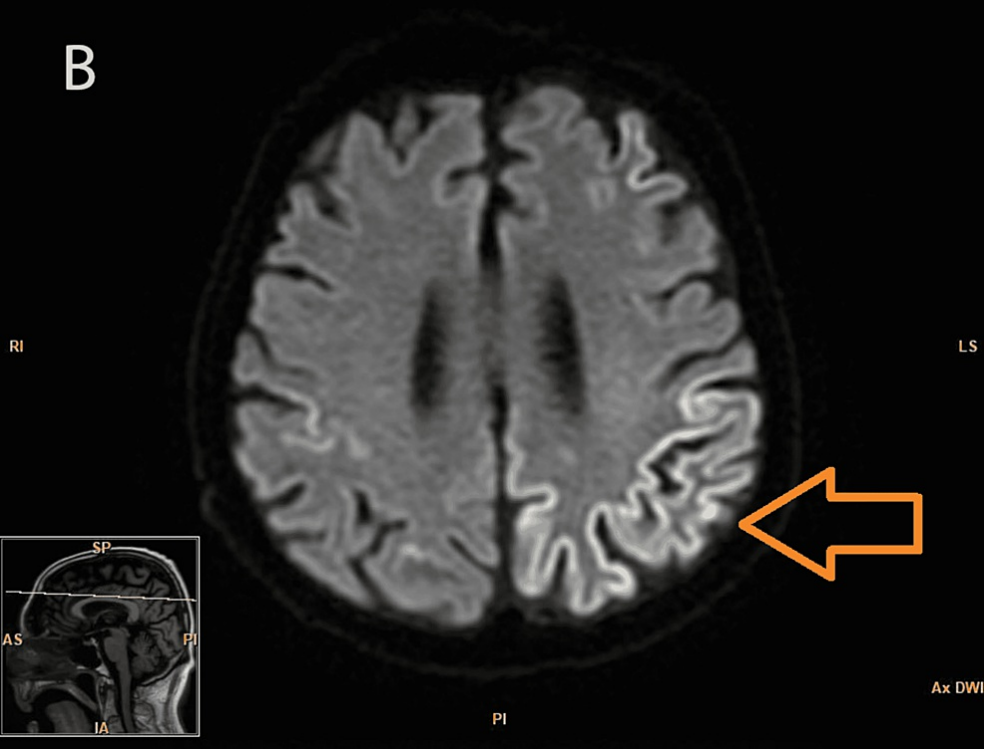
Axial diffusion-weighted MRI showing hyperintensities Abnormal high signal intensities in the occipital and parietal regions of the cortex (arrow)

On initial evaluation, his presentation was suggestive of rapid-onset dementia, likely subcortical in nature, for which he was started on a Sinemet (Levodopa + Carbidopa) trial. He was also evaluated for rapidly progressing dementia with brain MRI with and without contrast. The MRI of the brain was done approximately two weeks after the presentation (Figures 1-2), which showed bilateral gyriform high signal intensities (arrow) in the occipital and parietal regions on diffusion-weighted imaging (DWI) and fluid-attenuated inversion recovery (FLAIR) imaging.

Electroencephalogram (EEG) showed nonspecific slow-wave abnormalities; lumbar puncture showed cerebrospinal fluid (CSF) neuron-specific enolase was elevated at 44 (abnl > 30); real-time quaking-induced conversion (RT qUIC) CSF test came back positive; T-tau protein CSF was elevated at 2155 (normal range: 0-1149 pg/ml); and 14-3-3 protein in CSF was also elevated at 13,114 arbitrary units (AU)/ml (0-1999 AU/ml). The patient met probable criteria for sporadic CJD on account of rapidly progressive dementia, with extrapyramidal signs, positive RT qUIC CSF, positive 14-3-3 CSF assay, and high signal in the occipital and parietal regions bilaterally on DWI and FLAIR imaging.

## Discussion

CJD is classified as sporadic (sCJD), genetic (gCJD), acquired (iatrogenic (iaCJD), and variant (vCJD)). About 85% to 95% of all cases of CJD are sporadic, the rest include 5% to 15% of gCJD cases, and less than 1% of iaCJD and vCJD cases [7] (Table 1).

**Table 1:** Etiological classification of Creutzfeldt–Jakob disease

Type	Disease
Sporadic or idiopathic	Sporadic Cruetzfeldt–Jakob disease
	Sporadic fatal insomnia
	Variably protease-sensitive prionopathy
Genetic (PRNP gene)	Genetic CJD
	Gerstmann-Strausller-Scheinker
	Familial Fatal insomnia
Acquired	Kuru
	Iatrogenic CJD
	Variant CJD

The mean age for the onset of the disease is about 62 years, even though uncommon instances in young adults and people over 80 years of age have been reported [8,9]. Generally, sporadic CJD patients have a very short disease course, lasting only five months or less, although some factors such as age and PRNP genotype can influence the disease course [5]. Alcohol dependence poses a diagnostic problem in the diagnosis of CJD. Only a few reports have been published on the comorbidity of alcohol use disorder and CJD, specifically CJD associated with Wernicke encephalopathy [10], CJD manifesting Wernicke-Korsakoff syndrome [11,12], and also Wernicke encephalopathy manifesting variant CJD [13]. The onset of early CJD symptoms can be challenging to detect. Our patient presented with a slow gait, tremors in the upper limbs, and frequent falls, which were attributed to alcohol use and were interpreted as alcohol-related psychoses and withdrawal. Although these can be attributed to alcohol-related conditions, we cannot convincingly rule out other diagnoses, including CJD, as presented in this report.

Diagnosis

The presence of rapidly progressive dementia syndromes, especially those associated with myoclonus, ataxia, or visual disturbances, should raise suspicion of CJD. Other potentially treatable disorders should be excluded (Table 2). CJD is primarily diagnosed by a neuropathologic examination detecting protease-resistant PrPSc (PrPres), which is performed by obtaining a brain biopsy, but sCJD can also be detected noninvasively. A thorough history and neuropsychiatric examination with specific diagnostic tests including EEG, MRI, and tests for CSF protein markers such as RT-QuIC, neuron-specific enolase, T tau protein, and 14-3-3 protein can aid in the premortem diagnosis of sCJD. Restricted diffusion is apparent in at least two cortical regions or is predominant in the caudate nucleus, putamen, and thalamus on MRI in patients with sCJD. MRI generally has better diagnostic accuracy than T tau and 14-3-3 CSF protein testing [1,14,15].

Real-time quaking-induced conversion test (RT-QuIC)

As a protein amplification assay, RT-QuIC is the preferred choice for clinical testing because of its ease of standardization, quicker turnaround time, and lack of infectious byproducts. Inducing pathologic seeding using shaking rather than sonication, amplifies the PrP levels [16]. In addition to cerebrospinal fluid (CSF), olfactory epithelium, skin, ocular tissue, and other types of tissues can be used in RT-QuIC testing [17-20]. RT-QuIC testing was noted to have 92-95% sensitivity and 98.5-100% specificity in a United States sample [21]. CDC diagnostic criteria for sCJD are enumerated in Table 2 [22].

**Table 2:** Sporadic Creutzfeldt-Jakob Disease diagnostic criteria by CDC, 2018 RT-QuIC: real-time quaking-induced conversion; CSF: cerebrospinal fluid; EEG: electroencephalogram; DWI: diffusion-weighted imaging; FLAIR: fluid-attenuated inversion recovery [22]

Sporadic CJD	
Definite diagnosis	Neuropathological confirmation
Probable diagnosis	Neuropsychiatric disorder with a positive RT-QuIC test in CSF or other tissues
	OR
	Rapidly progressive dementia and at least two out of the following four clinical features: myoclonus, cerebellar or visual signs, pyramidal or extrapyramidal signs, akinetic mutism
	AND
	One or more of the following tests should be positive: EEG with periodic sharp wave complexes, positive 14-3-3 CSF assay with a duration of disease <2 years, MRI findings of hyperintensities in caudate/putamen and/or in at least two cortical regions (temporal, parietal, occipital) either on DWI or FLAIR
	AND
	With no alternative diagnosis indicated by routine investigations

There are many other neurological disorders that can mimic prion disease and need to be ruled out before we entertain the diagnosis of prion disease. The diseases that mimic prion disease are broad and are enumerated in Table 3 [2].

**Table 3:** Differential diagnosis

Diagnosis	Feature
Alzheimer's disease	Diffuse cortical atrophy seen on MRI or CT, specifically in hippocampal or temporal regions. Similar atrophy is often minimal or absent in sCJD.
	MRI of sCJD patients often shows increased signal intensities in bilateral putamen and caudate nuclei.
Dementia with Lewy bodies	Less rapid course compared to sCJD.
	Early prominent feature is visual hallucinations.
	Patients with dementia with Lewy bodies do not show hyperintensities in the putamen or caudate nuclei on MRI.
Frontotemporal dementia	Early prominent personality and behavior changes.
	Frontal and/or temporal lobe atrophy is typically seen on MRI.
Meningoencephalitis	Symptoms of confusion, headache, fever, and seizure usually with acute onset.
	Memory impairment takes a rapid course than in sCJD.
	Inflammatory markers such as increased white cell count or protein in CSF examination are seen which are absent in sCJD.
Alcohol use disorder	Wernicke’s encephalopathy: encephalopathy, oculomotor dysfunction, and gait instability.
	Korsakoff’s psychosis: occurs after an episode of Wernicke’s encephalopathy, presents with selective antegrade and retrograde amnesia.
	Marchiafava–Bignami disease: dementia can be acute, dysarthria, spasticity, and gait abnormalities are classical presenting symptoms.
	Alcoholic cerebellar degeneration: unsteady gait, poor coordination, memory impairment.
Corticobasal degeneration	Disease course is of longer duration and slowly progressive typically around 10 years and typical signs include dysphasia, limb apraxia, myoclonus, etc.
Paraneoplastic encephalomyelitis	Often associated with malignancy
	Inflammatory markers in the CSF are typically seen.
	Anti-Hu antibodies are usually seen in paraneoplastic encephalomyelitis.
Progressive supranuclear palsy	Vertical gaze abnormalities and postural instability are early findings.
Cerebral autosomal dominant arteriopathy with subcortical infarcts and leukoencephalopathy (CADASIL)	Earlier onset
	MRI shows characteristic multiple frontal lobe hyperdensities in periventricular white matter.
	Positive family history is present.

As of now, there are no effective treatments available for human prion diseases [23]. Current care entails supportive treatment [24]. There have been numerous strides in exploring investigational treatments for human prion diseases, and further research is underway. A non-opioid analgesic with a central acting mechanism, flupirtine maleate, is known to have cytoprotective effects in vitro in neurons inoculated with prion protein fragments [25]. A heparin mimetic, pentosan polysulfate (PPS), interferes with the conversion of PrPC to PrPSc, thus altering the disease mechanism [26]. A cultured neuroblastoma cell line (ScN2a) chronically infected with prions was inhibited in its formation of PrPSc by quinacrine and chlorpromazine, particularly derivatives of acridine and phenothiazine [27]. It is also hypothesized that minocycline and doxycycline prevent protein misfolding by the pathologic form of the prion protein in in-vitro and animal models [28]. However, further validation of these treatments needs to be done in the clinical setting.

## Conclusions

Sporadic CJD is an uncommon disease that presents with rapid onset dementia and, unfortunately, has a poor prognosis. Its overlapping presentation with other commonly occurring diseases such as Alzheimer's disease, Lewy body dementia, and especially alcohol use disorders, may result in delayed diagnosis. Early signs tend to be non-specific with subjective complaints that can be attributed to alcohol-related psychosis. In such cases, identifying when the symptoms of CJD began can pose a challenge. Hence, a high index of suspicion is necessary. Fortunately, advances in immunological and biochemical testing now allow for less invasive diagnostic testing of CJD with reasonable certainty as compared to the gold standard testing with brain biopsy. Furthermore, despite CJD being a rare entity, it can be a reasonable differential when diagnosing alcohol use disorders. Our case serves to add value to this rare diagnostic dilemma of CJD and alcohol use disorder.
